# Effectiveness and residual speed of flea kill of a novel spot on formulation of spinetoram (Cheristin^®^) for cats

**DOI:** 10.1186/s13071-017-1996-9

**Published:** 2017-02-02

**Authors:** Tandy Paarlberg, Joseph Winkle, Anthony J. Rumschlag, Lisa Marie Young, William G. Ryan, Daniel E. Snyder

**Affiliations:** 1 Elanco Animal Health, 2500 Innovation Way, Greenfield, IN USA; 2 Ryan Mitchell Associates, LLC, Westfield, NJ USA

**Keywords:** Cats, Fleas, Spinetoram, Cheristin, Imidacloprid, Fipronil

## Abstract

**Background:**

A spot-on spinetoram formulation (Cheristin^®^) was developed to eliminate fleas from infested cats. This paper describes three spinetoram studies: two for registration (Studies 1 and 2), and one comparing residual speed of kill (SOK) with topically applied fipronil/(S)-methoprene (FSM) and imidacloprid (Study 3).

**Methods:**

Cats were randomized to treatment based on flea counts from infestations placed within 2 weeks prior to treatment. In Studies 1 and 2, groups were untreated control and spinetoram; in Study 3, groups were untreated control, spinetoram, FSM and imidacloprid, all applied per label on Day 0. Cats were infested the day before treatment. In Studies 1 and 2, counts were completed 48 h post-treatment and after weekly challenges through 5 weeks. In Study 3, infestations were completed weekly through Day 28, with counts 1, 4, 8 and 12 h after treatment or post-infestation (PI). Efficacy was determined on geometric mean flea count reductions compared with controls, and in Study 3 mean flea counts in spinetoram-groups were compared with those in FSM and imidacloprid groups.

**Results:**

In Studies 1 and 2, spinetoram effectiveness was 100% against existing infestations, and at least 96% through Day 37. In Study 3 mean counts were not significantly different from controls in any group until 8 h post-treatment when imidacloprid counts were significantly lower than spinetoram counts, which were in turn significantly lower than FSM counts (*P* < 0.05). At 1 h PI spinetoram-group counts were significantly lower (*P* < 0.05) than counts in: controls, all days; imidacloprid, Days 7, 14, and 28; FSM, Days 14 and 28. At 4 h PI, spinetoram mean counts were significantly lower (*P* < 0.05) relative to: controls, all days; imidacloprid, Days 7, 14 and 21; FSM, Days 7, 14, 21 and 28 (*P* < 0.05). On multiple occasions, at 8 and 12 h PI, mean counts were significantly lower (*P* < 0.05) for spinetoram than for imidacloprid and FSM; at no point were FSM or imidacloprid significantly more effective than spinetoram against new infestations. All treatments were well tolerated.

**Conclusions:**

Spinetoram was highly effective for at least 1 month post-treatment and provided more rapid month-long residual SOK than FSM or imidacloprid.

## Background

The spinosyns are a family of insecticidal fermentation products, derived from the actinomycete, *Saccharopolyspora spinosa*. These compounds exert their selective activity against insects primarily by allosterically activating nicotinic acetylcholine receptors (nAChRs), binding at receptor sites distinct from those at which other insecticides exert their activity. Some activity is also provided by spinosyns binding to γ-amino-butyric acid (GABA) receptors with an overall effect that leads to rapid insect paralysis and death [1–3]. The first of these compounds to be used to treat ectoparasitic infestations of animals was spinosad, which in different countries has approvals for use in or on cattle, sheep, dogs and cats, and also as a head lice treatment for humans.

The large and complex chemical structure of the spinosyns presented opportunities for modifications to enhance their chemical properties and biological activity. The outcome of the application of artificial neural networks to quantitative structure activity relationships was the discovery of spinetoram, a mixture of major (3’-ethoxy-5,6-dihydro spinetoram J) and minor (3’-ethoxy spinetoram L) components. Testing in agriculture systems demonstrated that spinetoram maintained the exceptional environmental and toxicological profile already established for the spinosyn class of chemistry [4]. Like spinosad some years beforehand, spinetoram received the United States Presidential Green Chemistry award for 2008 (Environmental Protection Agency).

Anecdotal reports of the unreliable effectiveness of common topically applied products for cats were substantiated in a study in which three consecutive monthly treatments of client-owned cats with fipronil produced only a 46.1% reduction from baseline in geometric mean flea counts. In that study, an early formulation of spinetoram administered according to the same schedule produced geometric mean flea count reductions of 99.4% [5]. Although that 39.6% w/v formulation of spinetoram was non-irritant, some field reports were received of focal hair loss from the application site in a small percentage of treated cats. This problem was attributed to the high viscosity of the formulation and so a formulation with a lower concentration and viscosity was developed [6]. Preliminary laboratory effectiveness studies were completed, and tolerability studies in client-owned cats demonstrated the acceptability and safety of an 11.2% formulation (unpublished observations).

This report describes three studies undertaken to determine the effectiveness of this 11.2% spinetoram spot-on formulation (Cheristin^®^, Elanco, Greenfield, IN) against fleas on cats. Two studies (Studies 1 and 2) determined effectiveness when flea (*Ctenocephalides felis*) counts were completed at 48 h post-treatment, and at 48 h post-infestations applied over the 5 weeks after treatment. Study 3 focused on the residual speed of kill of spinetoram compared with that provided by imidacloprid (Advantage^®^ II, Bayer Animal Health, Overland Park, KS) and by fipronil/(*S*)-methoprene (Frontline^®^ Plus, Merial, Atlanta, GA).

## Methods

### Design of studies

All studies were controlled and utilized a blinded, randomized complete block design with pre-treatment live flea counts as a blocking factor. All protocols were approved by the relevant Institutional Animal Care and Use Committees. Uniquely identified domestic short-hair/cross bred cats, at least 7 months of age and weighing between 2.2 and 6.6 kg were acclimated in the trial facility for at least 14 days before the study began, and throughout each study cats were maintained in separate individual cages. Pre-treatment flea challenges (approximately 100 fleas per cat) and subsequent flea comb counts 48 h post-challenge were made at least once in each study, from 9 days to 7 days prior to treatment. These counts were used to verify that cats selected for the study would carry a burden of at least 30 adult fleas.

Cats meeting the inclusion/exclusion criteria with the highest counts were rank-ordered from highest to lowest. Blocks were formed by assigning the appropriate number of cats, based on the number of treatment groups, with the highest *C. felis* counts to the first block, the same number of cats with the next highest counts to the second block, and so on until the protocol-designated number of blocks was defined. Treatments were randomly assigned to cats within each block. Cats were housed individually in indoor stainless-steel cages. Treated cats were not housed over control cats, or over other treatment group cats where banks of cages (top/bottom arrangement) were used. In all three studies, the primary objective was the percent reduction in geometric mean live flea counts in treated groups when compared with counts from untreated or sham-treated control cats.

### Treatments and groups

In all studies, treatments were administered once, on Day 0. In Studies 1 and 2 there were 8 cats per group, and in Study 3 six cats per group for each assessment point.

In Studies 1 and 2 cats were randomized among two treatment groups: Group 1: sham-treated topically with product vehicle without active ingredient (negative control); and Group 2: treated topically with the spinetoram 11.2% formulation (Cheristin).

In Study 3, 96 cats were randomized among four treatment groups, with three groups to receive commercially available formulations of topically applied products, all administered according to label: Group 1: Spinetoram 11.2% (Cheristin; Elanco); Group 2: Fipronil 9.8% w/v and (S)-methoprene 11.8% w/v (Frontline Plus for Cats); Group 3: Imidacloprid 9.1% w/v and pyriproxyfen 0.46% (Advantage II); Group 4: Untreated control.

Each treatment group was subdivided into 4 sub-groups, each of six cats, formed by ranking on the basis of Day -7 flea counts and randomizing to times of flea counts following treatment and after each subsequent flea infestation.

### Flea infestations and counts

In all studies flea infestations were completed on Day -1, and then at weekly intervals from Days 7 to 28. In Studies 1 and 2 an additional infestation was completed on Day 35.

In Studies 1 and 2, flea counts were completed 48 h post-treatment and 48 h after each subsequent infestation. In Study 3, flea counts were completed 1, 4, 8 and 12 h after treatment and after each post-treatment infestation on Days 7, 14, 21 and 28. In all 3 studies, fleas removed during each flea count time point were not put back onto the cats. The effectiveness calculation for each product was based on reductions in mean live flea counts at 1, 4, 8 and 12 h after treatment or after subsequent infestations compared to mean live flea counts in untreated control cats infested according to the same schedule.

All flea infestations were completed using each facility’s in-house flea colonies which are periodically refreshed by the introduction of locally caught fleas. The infestation procedure involved placement of approximately 100 unfed, adult cat fleas onto each cat’s dorsum from the nuchal crest or shoulder area to the lumbar-sacral region, with the cat gently restrained in a standing or sternal recumbency position. Post-treatment infestations were made to avoid placing fleas near the area of highest insecticidal concentration. Live flea counts on each cat were obtained by thorough combing using a metal flea comb for a minimum of 5 min. If no fleas were found in this time, an additional 10 strokes of the flea comb were performed over each area of the body - dorsal, ventral, legs and thorax. If a flea was recovered, the 10-stroke routine was repeated until no fleas were recovered in any area. Care was taken to avoid the site of test substance application during the first 24 h after treatment. Combs were cleaned with alcohol after each use, and combs used for cats in the same treatment group were not used on cats in other treatment groups.

### Efficacy assessments

Geometric mean counts were used to assess the primary objective of reductions in flea counts when treated groups were compared to controls. Percent efficacy at each time point was calculated using the formula:

$$ \%\ \mathrm{Efficacy} = \left(\mathrm{Mean}\ \mathrm{control}\ \mathrm{counts}\ \hbox{--}\ \mathrm{Mean}\ \mathrm{treated}\ \mathrm{cat}\ \mathrm{counts}\right) \times 100/\ \mathrm{Mean}\ \mathrm{control}\ \mathrm{counts} $$

Logarithmic transformation of flea counts was performed using the logarithm of flea count + 1 to account for zero values. Geometric mean values were obtained *via* back-transformation of transformed mean counts and subtracting one. Arithmetic means were also calculated.

For each time point, repeated measures analysis of variance was generated. The model included treatment group, study day and treatment group by study day interaction as fixed effects and case id as a random effect. The percent reduction compared to control was obtained from the least squares means of each model. Pairwise comparisons between all treatment groups was also obtained from these models. Primary objective assessments were based on geometric mean count reductions.

## Results

All treatments were well tolerated in each study and, other than for occasional mild and transient cosmetic changes typical of products applied topically to cats (e.g. hair clumping), there were no treatment-related adverse events. Post-treatment application-site observations indicated that any wetness and/or hair clumping in spinetoram-treated cats on the day of, or the day after treatment had disappeared during the following days.

**Fig. 1:**
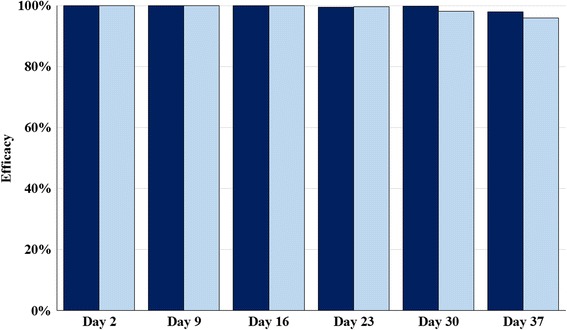
Percentage reduction in geometric mean flea counts in spinetoram-treated cats from weekly post-treatment challenge infections through Day 42 (counts 48 h post-treatment and after each infestation). *Key*:* dark blue*, Study 1; *light blue*, Study 2

In Studies 1 and 2, when flea counts were completed 48 h post-treatment and after each subsequent infestation, the percentage effectiveness was 100% against infestations present at the time of treatment, with at least 96% effectiveness based on geometric mean counts maintained through Day 37 (Tables 1 and 2; Fig. 1). The least squares mean differences between the transformed flea counts in the treated and control groups were statistically significant (*P* < 0.0001) for each study day.

**Table 1:** Geometric and arithmetic mean (standard deviation) flea counts of spinetoram-treated cats and percent reduction relative to geometric mean counts in control cats in Study 1

	Day of study					
	2	9	16	23	30	37
Untreated Control						
Arithmetic mean	87.0 (10.9)	76.4 (17.2)	77.6 (15.3)	78.0 (11.3)	59.9 (21.2)	61.3 (17.0)
Geometric mean	86.4	74.5	76.4	77.2	56.3	59.3
Spinetoram						
Arithmetic mean	0 (0)	0 (0)	0 (0)	0.8 (1.2)	0.1 (0.4)	2.0 (2.5)
Geometric mean	0*	0*	0*	0.5*	0.1*	1.3*
% Reduction from Control	100	100	100	99.4	99.8	97.9

*P < 0.0001

**Table 2:** Geometric and arithmetic mean (standard deviation) flea counts of spinetoram-treated cats and percent reduction relative to geometric mean counts in control cats in Study 2

	Day of study					
	2	9	16	23	30	37
Untreated Control						
Arithmetic mean	56.1 (11.1)	67.1 (15.0)	65.6 (11.8)	61 (18.8)	73.4 (18.2)	68.3 (11.3)
Geometric mean	57.0	65.5	64.7	58.1	71.2	67.4
Spinetoram						
Arithmetic mean	0 (0)	0 (0)	0 (0)	0.4 (1.1)	2.1 (2.3)	7.1 (10.2)
Geometric mean	0.0*	0.0*	0.0*	0.2*	1.4*	2.7*
% Reduction from Control	100	100	100	99.7	98.1	96.0

*P < 0.0001

In Study 3, the comparative speed of flea kill study, no product demonstrated significant reductions from the control group in mean flea counts at 1 and 4 h post- treatment (Day 0) (Tables 3 and 4). At the 1-h post-infestation time point only the spinetoram group showed a significant reduction from controls on Day 7 (*t*-test: *t*_(80)_ = -3.03, *P* = 0.0033), when mean flea counts were significantly lower than in the imidacloprid group (*t*-test: *t*_(80)_ = -2.06, *P* = 0.0428) (Table 3). At this 1-h time point the spinetoram group also had significantly lower mean counts than the imidacloprid group on Days 14 (*t*-test: *t*_(80)_ = -5.19, *P* < 0.0001), and 28 (*t*-test: *t*_(80)_ = -3.64, *P* = 0.0005), and the fipronil/(S)-methoprene group on Days 14 (*t*-test: *t*_(80)_ = -3.85, *P* = 0.0002), 21 (*t*-test: *t*_(80)_ = -3.01, *P* = 0.0035) and 28 (*t*-test: *t*_(80)_ = -2.58, *P* = 0.0116). At 4 h post-infestation, spinetoram provided significantly greater effectiveness than imidacloprid on Days 7 (*t*-test: *t*_(80)_ = -2.93, *P* = 0.0044), 14 (*t*-test: *t*_(80)_ = -2.10, *P* = 0.0388) and 21 (*t*-test: *t*_(80)_ = -3.21, *P* 0.0019) and fipronil/(S)-methoprene on Days 7 (*t*-test: *t*_(80)_ = -5.83, *P* < 0.0001), 14 (*t*-test: *t*_(80)_ = -6.18, *P* < 0.0001), 21 (*t*-test: *t*_(80)_ = -5.54, *P* < 0.0001) and 28 (*t*-test: *t*_(80)_ = -2.76, *P* = 0.0072) (Table 4).

**Table 3:** Geometric and arithmetic mean (standard deviation) flea counts 1 h post-treatment (Day 0) and post-reinfestations in Study 3

	Day 0	Day 7	Day 14	Day 21	Day 28
Untreated Control					
Arithmetic mean	55.3 (12.7)	68.5 (9.7)	69.7 (12.6)	81.2 (7.2)	86.0 (7.2)
Geometric mean	54.2	68.0^a^	68.6^a^	80.9^a^	85.6^b^
Imidacloprid					
Arithmetic mean	63.8 (11.4)	60.6 (6.0)	55.2 (13.8)	59.5 (17.4)	65.5 (9.0)^b,d^
Geometric mean	62.9	60.2^d^	53.6^c^	57.6^a^	65.0^b,d^
% Reduction from Control	0	11.4	21.9	28.9	24.1
Fipronil/(S)-methoprene					
Arithmetic mean	70.2 (13.3)	55.3 (13.4)	45.7 (5.9)	66.3 (8.3)	57.5 (9.2)
Geometric mean	69.1	53.8	45.4^a,b^	65.9^c^	57.0^b,d^
% Reduction from Control	0	20.8	33.9	18.5	33.5
Spinetoram					
Arithmetic mean	62.0 (17.5)	47.5 (9.8)	30.5 (14.4)	45.5 (4.3)	42.3 (9.9)
Geometric mean	60.1	46.6^a,d^	28.0^a,c^	45.3^a,c^	41.3^b,d^
% Reduction from Control	0	31.4	59.2	44.0	51.8

*Note*: Numbers within columns with the same superscript are significantly different: Treated group comparison with control: ^a^ *P* < 0.01; ^b^ *P* < 0.05; Spinetoram comparison with imidacloprid or fipronil/(S)-methoprene: ^c^ *P* < 0.01; ^d^ *P* < 0.05

**Table 4:** Geometric and arithmetic mean (standard deviation) flea counts 4 h post-treatment (Day 0) and post-reinfestations in Study 3

	Day 0	Day 7	Day 14	Day 21	Day 28
Untreated Control					
Arithmetic mean	58.5 (12.5)	73.2 (6.6)	73.8 (15.0)	68.2 (14.1)	71.8 (12.1)
Geometric mean	57.2	72.9^a^	72.4^a^	67.0^b^	70.9^c^
Imidacloprid					
Arithmetic mean	40.2 (11.4)	28.3 (17.4)	13.8 (6.9)	23.3 (9.3)	39.0 (14.2)
Geometric mean	38.1	24.3^a,c^	12.5^a,d^	21.2^b,c^	36.3^c^
% Reduction from Control	33.4	66.7	82.7	68.4	48.8
Fipronil/(S)-methoprene					
Arithmetic mean	45.7 (6.1)	50.5 (5.5)	38.0 (14.9)	39.5 (12.5)	53.0 (13.4)
Geometric mean	45.3	50.3^c^	35.4^a,d^	38.0^b,c^	51.3^c^
% Reduction from Control	20.7	31.1	51.1	43.2	27.7
Spinetoram					
Arithmetic mean	49.8 (17.5)	13.0 (7.3)	10.0 (7.9)	10.0 (4.3)	28.0 (12.2)
Geometric mean	46.5	11.4^a,c^	7.1^a,d^	9.2^b,c^	25.8^a,c^
% Reduction from Control	18.6	84.3	90.2	86.3	63.7

*Note*: Numbers within columns with the same superscript are significantly different: Treated group comparison with control: ^a^ *P* < 0.01; ^b^ *P* < 0.05; Spinetoram comparison with imidacloprid or fipronil/(S)-methoprene: ^c^ *P* < 0.01; ^d^ *P* < 0.05

At 8 h post-treatment, only the fipronil/(S)-methoprene group failed to achieve significant reductions in mean flea counts from the control group. Mean counts in the spinetoram group were significantly lower (*P* < 0.05) than in the fipronil/(S)-methoprene group but significantly higher (*P* < 0.05) than in the imidacloprid group (Table 5). For all counts completed 8 h after each infestation timepoint, mean flea burdens in the spinetoram group were significantly lower than in the control group (Day 7: *t*-test: *t*_(80)_ = -5.60, *P* < 0.0001; Day 14: *t*_(80)_ = -8.62, *P* < 0.0001; Day 21: *t*_(80)_ = -5.58, *P* < 0.0001; Day 28: *t*_(80)_ = -4.36, *P* < 0.0001) and significantly lower than in the fipronil/(S)-methoprene group on Days 7 (*t*-test: *t*_(80)_ = -2.61, *P* = 0.0108), 14 (*t*-test: *t*_(80)_ = -4.37, *P* < 0.0001) and 28 (*t*-test: *t*_(80)_ = -2.52, *P* = 0.0138).

**Table 5:** Geometric and arithmetic mean (standard deviation) flea counts 8 h post-treatment (Day 0) and post-reinfestations in Study 3

	Day 0	Day 7	Day 14	Day 21	Day 28
Untreated Control					
Arithmetic mean	67.3 (6.7)	71.5 (9.2)	70.5 (13.4)	74.7 (11.3)	71.5 (6.1)
Geometric mean	67.1^a^	71.0^a^	69.4^a^	73.9^a^	71.3^a^
Imidacloprid					
Arithmetic mean	11.3 (8.8)	10.3 (7.6)	8.5 (9.3)	16.5 (8.2)	29.0 (10.9)
Geometric mean	8.9^a,d^	8.3^a^	4.7^a^	14.4^a^	27.1^a^
% Reduction from Control	86.8	88.3	93.2	80.5	62.0
Fipronil/(S)-methoprene					
Arithmetic mean	53.8 (18.8)	29.5 (16.0)	18.5 (10.9)	13.0 (6.3)	38.2 (11.8)
Geometric mean	51.0^d^	23.9^a,d^	14.6^a,c^	11.9^a^	36.6^d^
% Reduction from Control	24.0	66.3	78.9	83.9	48.7
Spinetoram					
Arithmetic mean	26.8 (18.1)	10.5 (7.3)	3.2 (2.8)	10.5 (6.2)	19.3 (15.7)
Geometric mean	21.4^a,d^	8.9^a,d^	2.3^a,c^	9.4^a^	14.4^a,d^
% Reduction from Control	68.1	87.5	96.7	87.3	79.8

*Note*: Numbers within columns with the same superscript are significantly different: Treated group comparison with control: ^a^ *P* < 0.01; ^b^ *P* < 0.05; Spinetoram comparison with imidacloprid or fipronil/(S)-methoprene: ^c^ *P* < 0.01; ^d^ *P* < 0.05

At 12 h post-treatment and on all days post-infestation, flea counts in all treated groups were significantly lower than those of the control groups. At 12 h post-treatment, mean flea counts in the imidacloprid and fipronil/(S)-methoprene groups were significantly lower than those of the spinetoram group (*t*-test: *t*_(80)_ = 2.29, *P* = 0.0245 and *t*_(80)_ = 2.19, *P* = 0.0315, respectively) (Table 6). At 12 h post-infestation mean flea counts in the spinetoram group were significantly lower than in the imidacloprid group on Days 7 (*t*-test: *t*_(80)_ = -2.55, *P* = 0.0126) and 21 (*t*-test: *t*_(80)_ = -3.21, *P* = 0.0019), and significantly lower than the fipronil/(S)-methoprene group on Day 21 (*t*-test: *t*_(80)_ = -2.49, *P* = 0.0148).

**Table 6:** Geometric and arithmetic mean (standard deviation) flea counts 12 h post-treatment (Day 0) and post-reinfestations in Study 3

	Day 0	Day 7	Day 14	Day 21	Day 28
Untreated Control					
Arithmetic mean	71.7 (16.4)	77.3 (9.2)	60.3 (10.4)	72.3 (15.7)	77.3 (10.8)
Geometric mean	70.2^a^	76.9^a^	59.6^a^	71.0^a^	76.7^b^
Imidacloprid					
Arithmetic mean	7.2 (6.2)	10.2 (6.7)	5.8 (4.6)	7.8 (3.0)	16.8 (9.3)
Geometric mean	4.7^a,d^	8.6^a,d^	3.9^a^	7.4^a,d^	14.8^b^
% Reduction from Control	93.3	88.9	93.4	89.6	80.8
Fipronil/(S)-methoprene					
Arithmetic mean	14.8 (19.8)	4.7 (6.6)	5.3 (9.8)	6.3 (5.6)	24.7 (11.6)
Geometric mean	5.0^a,d^	2.0^a^	2.0^a^	4.9^a,d^	21.7^b^
% Reduction from Control	92.9	97.4	96.6	93.1	71.7
Spinetoram					
Arithmetic mean	22.5 (17.1)	3.5 (4.7)	3.2 (3.5)	1.2 (1.6)	13.3 (12.9)
Geometric mean	16.4^a,d^	1.8^a,d^	2.1^a^	0.8^a,d^	8.6^b^
% Reduction from Control	76.6	97.7	96.5	98.9	88.8

*Note*: Numbers within columns with the same superscript are significantly different: Treated group comparison with control: ^a^ *P* < 0.01; ^b^ *P* < 0.05; Spinetoram comparison with imidacloprid or fipronil/(S)-methoprene: ^c^ *P* < 0.01; ^d^ *P* < 0.05

At no post-treatment time point was the residual speed of kill of fipronil/(*S*)-methoprene or imidacloprid significantly greater than that of spinetoram against new flea infestations. Percent effectiveness for spinetoram was greater than for fipronil/(S)-methoprene or imidacloprid at multiple post-treatment timepoints.

## Discussion

As a combination of two semi-synthetic spinosyns (spinosyns J and L), spinetoram shares a common fermentation background and mode of action with spinosad, a mixture of spinosyns A and D. Spinosad delivered orally to dogs has been shown in laboratory flea infestation studies to have a rapid onset of activity that is sustained over a month following treatment [7–9]. This effectiveness has translated to high levels of flea control on dogs under field conditions in which monthly treatment with spinosad provided significant improvement in flea control and in signs of FAD, including owner-scored pruritus, when compared with other topically applied products [5, 9–11]. Similar findings have been reported for an oral formulation of spinosad administered to client-owned cats [12]. The results of the studies presented in this paper validate the potential value that a single topical application of 0.7 ml of this spinetoram formulation can bring to flea control in cats.

The two registration studies reported here, in which counts were completed 48 h after treatment and after each new infestation, demonstrated that spinetoram eliminated virtually all (98 to 100%) fleas through the month following treatment, with efficacy of 96.0 to 97.9% still apparent 5 weeks after treatment. However, flea egg production may begin within 24 to 36 h of the first blood meal, with 25% of fleas having fed within 5 min of finding a feline host and 97% within 1 h [13, 14]. Thus while the 48-h count can demonstrate the potential value of reducing egg output, and with repeated monthly treatments potentially solve the long term flea infestation problem in a local environment, a faster speed of kill is desirable to accelerate the epidemiological cure, to alleviate the irritation caused by flea bites and to reduce the risk of transmission of the cestode, *Dipylidium caninum*, and disease causing organisms such as *Bartonella henselae*.

Given the excellent speed of kill that orally administered spinosad has demonstrated in laboratory studies by killing more than 50% of fleas infesting dogs within 1 h and achieving 100% efficacy within 4 h of treatment, it had been anticipated that the similarly acting spinetoram applied topically to cats would match this rapid onset of effectiveness [7]. In fact, none of the topical products in this study achieved such a rapid onset of efficacy, and only imidacloprid and spinetoram showed significant differences from control at 8 h post-treatment. It would therefore seem that an interval of 12 to 24 h after treatment may be needed for topical products to achieve complete effectiveness against flea burdens present on cats at the time of treatment, and that systemically acting products may have a more rapid onset of activity. Support for this is provided in a report in which systemically acting products, by more effectively reducing the amount of blood fleas consume before dying, appeared to affect fleas infesting cats more quickly than treatments that act topically [15].

There are two possible explanations, not necessarily mutually exclusive, for the speed of flea knockdown of topical products to be quicker against new infestations than against flea burdens already established at the time of treatment. First, fleas that have established on the cat, having already taken a blood meal, may be more robust and so do not succumb as quickly to contact insecticidal activity. Secondly, topically applied products may require some amount of time to disperse from the site of application to ensure that fleas are exposed to lethal concentrations that will provide a rapid residual speed of kill. Importantly, the results of this study demonstrate that the residual speed of flea kill of spinetoram, applied topically, was at least equal to and at multiple times greater than that of fipronil/(S)-methoprene or imidacloprid for at least 4 weeks following application.

## Conclusions

In conclusion, the results of the three studies reported here show that this 11.2% spot-on formulation of spinetoram provides a high level of flea killing effectiveness that lasts beyond 1 month, and that a rapid speed of flea kill is maintained through at least 1 month. The rapid month-long residual speed of kill was more sustained for spinetoram than for fipronil/(S)-methoprene or imidacloprid, and demonstrates that this novel spinetoram formulation addresses the need for rapidly acting products that can substantially and quickly help to reduce irritation arising from flea bites, and break the flea life cycle by killing fleas before egg laying can begin.

## Abbreviation

SOK: Speed of kill
